# Autosomal recessive *IL12RB1* mutation: A case report of a Sudanese child and his father

**DOI:** 10.3389/fimmu.2023.1135824

**Published:** 2023-03-31

**Authors:** Omaima Abdelmajeed, Muna Mohammed Dawoud Ali, Nahla Hashim Erwa, Alamin Mustafa, Yassin Abdelraheem Ahmed, Rogaia Hasap Alrasoul Ahmed, Hala Hamza Eltayeb Mohammed, Malaz Elsadeg Hassan, Monzir Ahmed, Shima Algam

**Affiliations:** ^1^ Department of Pediatrics, Omdurman Islamic University, Omdurman, Sudan; ^2^ Department of Pediatrics, Tropical and Infectious Diseases, College of Medicine, University of Science and Technology, Khartoum, Sudan; ^3^ Faculty of Medicine, University of Khartoum, Khartoum, Sudan; ^4^ Faculty of Medicine, Al-Neelain University, Khartoum, Sudan; ^5^ Faculty of Medicine, Omdurman Islamic University, Omdurman, Sudan

**Keywords:** immunology, bacille Calmette–Guérin (BCG) vaccine, Mendelian susceptibility to mycobacterial diseases, tuberculosis, *IL12RB1* mutation

## Abstract

**Introduction:**

Mendelian susceptibility to mycobacterial disease (MSMD) is a rare inherited condition characterized by selective susceptibility to weakly virulent mycobacteria, such as substrains of the bacille Calmette–Guérin (BCG) vaccine and different environmental mycobacteria.

**Case presentation:**

A 7-year-old Sudanese boy was referred to the immunology clinic with a suspected diagnosis of MSMD. This followed multiple presentations with disseminated tuberculosis and typhoid fever. Genetic testing surprisingly revealed pathogenic homozygous variants in *IL12RB1* Exon 9, c.913A>T (p. Lys305*) in both the patient and his father, with a completely healthy asymptomatic carrier mother who is not blood related to the patient’s father.

**Conclusion:**

It is challenging to diagnose MSMD, especially in developing countries where health systems are poor and have limited resources. Family history and genetic tests may help in early MSMD treatment and avoiding disease complications.

## 1 Introduction

Inborn errors of immunity, also known as primary immunodeficiencies, are a group of rare genetic disorders characterized by a compromised immune system. These disorders often result in increased susceptibility to infections and other immunological problems (1). One of the key regulatory pathways in the immune system is the interferon-gamma (IFN-γ)-interleukin (IL)12/23 circuit, which is crucial for the development and function of T cells and the production of IL-12. IL-12 is an important cytokine that stimulates the immune response against intracellular pathogens, such as bacteria and viruses (2). Understanding the IFN-IL12/23 circuit and its role in primary immunodeficiencies is crucial for the diagnosis and management of disorders leading to increased susceptibility to mycobacterial disease.

Mendelian susceptibility to mycobacterial disease (MSMD) is a rare inherited condition characterized by selective susceptibility to weakly virulent mycobacteria, such as substrains of the bacille Calmette–Guérin (BCG) vaccine and different environmental mycobacteria (3). Many patients diagnosed with MSMD have invasive infections caused by other intra-macrophagic microorganisms, such as *Salmonella*, or mucocutaneous infections caused by *Candida* species (3–5). Because of incomplete penetrance and genetic differences, the presentation of MSMD is variable. Severe forms of MSMD cause early-onset, disseminated, and persistent life-threatening mycobacterial infections, while the milder forms may have a late onset, improve with age, or even remain clinically silent (3, 5, 6). Almost 20 genes are known to cause 34 different forms of MSMD. Most of these defects affect the IL-12/IFN-γ pathway proteins with the exception of *CYBB* and *ZNFX1* defects that affect the oxidative burst and the recruitment of stress granules, respectively (7). *CYBB* is a gene that regulates the respiratory burst in myeloid cells, including not only macrophages but also neutrophils. The Nicotinamide Adenine Dinucleotide Phosphate Oxidase, which plays a crucial role in the respiratory burst, is primarily located in neutrophils. This highlights the important role of *CYBB* in regulating cellular defense mechanisms in myeloid cells, particularly in response to pathogens and other foreign invaders (8, 9). The most frequent genetic cause of MSMD is *IL12RB1* deficiency (10).

## 2 Case presentation

**Figure 1:**
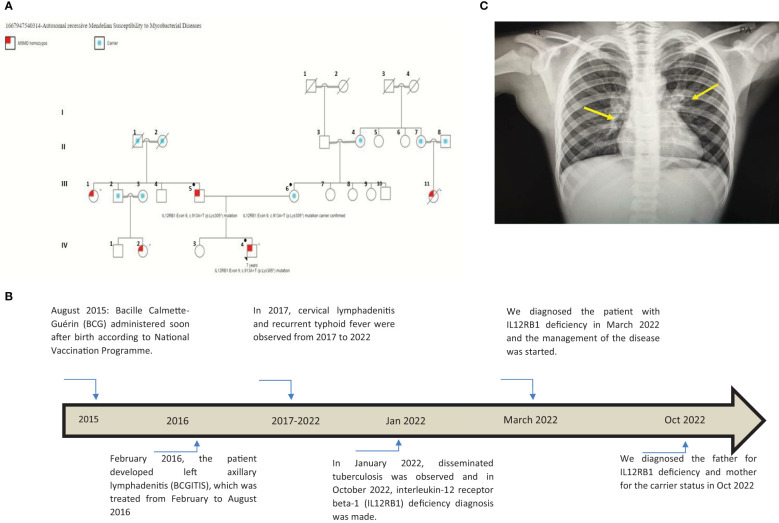
**(A)** This pedigree shows four generations of relations to the proband who is indicated with the black arrowhead IV-4. Red color in III-1, III-5, III-11, IV-2, and IV-4 indicates individuals affected by symptoms. Only the father (III-5), the mother (III-6), and the patient (IV-4) received genetic testing (indicated by ● in the pedigree); other affected individuals had clinical presentations highly suggestive of Mendelian susceptibility to mycobacterial disease (MSMD) and a history of multiple recurrent tuberculosis infections in childhood. III-7, III-8, III-9, III-10, and their offspring (not shown) have no MSMD symptoms or complaints. This pedigree was constructed using https://www.progenygenetics.com. **(B)** This figure illustrates the timeline of care for the patient (IV-4), with relevant date points highlighted, and identifies the major point of care. **(C)** This photographic image shows the chest x-ray for the patient, which is showing bilateral prominent hilar lymph nodes and bilateral diffuse reticulation with multiple hyperlucencies involving bilateral upper, middle, and left lower lobes (yellow arrows). These chest x-ray findings indicate early bronchiectasis.

A 7-year-old male patient (the proband, IV-4; **Figure 1A** **)** had a history of two episodes of mycobacterial lymphadenitis and recurrent typhoid fever. He was referred to the pediatric immunology clinic due to a newly diagnosed disseminated tuberculosis (TB) affecting the lungs and multiple lymph nodes that included the mesenteric lymph nodes. The patient had a history of fever and left axillary lymphadenitis at the age of 6 months, for which he received oral and injectable antibiotics without improvement. Excisional biopsy and histopathology showed caseating granuloma diagnosed as BCGitis, and he was treated with four anti-TB drugs (rifampicin, isoniazid, pyrazinamide, and ethambutol) for only 6 months. At 2 years of age, he developed fever, weight loss, and cervical lymphadenopathy. Fine-needle aspiration cytology showed caseating granuloma. He received the same combination of anti-TB drugs as before for 6 months, and his symptoms improved **(** **Figure 1B** **)**.

He also had recurrent febrile illnesses associated with abdominal pain and mesenteric lymphadenitis that were diagnosed as typhoid fever on many occasions based on a very high antibody titer on Widal test (culture not done) **(** **Figure 1B** **)**. On each occasion, his condition improved remarkably after treatment. There was no history of other infections (pneumonia, otitis media, nor skin, bone, or joint infections). The patient’s new TB condition started 6 weeks before referral to the Pediatric Tropical and Infectious Diseases unit. He appeared to have normal growth with a weight of 20 kg (10th) and a height of 118 cm (25th). He presented with high-grade fever, mainly at night with sweating, which improved with oral paracetamol. He also had severe abdominal pain disturbing his sleep and daily living activity, but there was no abdominal distension, diarrhea, constipation, or vomiting. The mother noted that the patient had significant weight loss and fatigability. Two weeks later, the patient developed a cough, which started dry and then became productive with thick mucoid sputum without blood. He did not suffer from chest pain or shortness of breath. In this occasion (January 2022), his diagnosis was disseminated TB **(** **Figure 1B** **)**. His family history was remarkable for the affection of four other members **(** **Figure 1A**: III-1, III-5, III-11, IV-2, and IV-4) across two generations including his father **(** **Figure 1A**: III-5), with a similar condition. The patient’s father **(** **Figure 1A**: III-5), who was 40 years old at the time of presentation of the proband, had recurrent TB adenitis since early childhood, which was diagnosed based on histopathology and acid-fast *Bacillus* (AFB) stain. Furthermore, the proband’s parents reported that the father had three episodes of meningitis. One of these was cerebrospinal fluid (CSF)-confirmed tuberculous meningitis. In two occasions, failure of therapy was followed by the detection of *Aspergillus* fungal hyphae on the CSF. As a result of complicated TB and fungal meningitis, the father is now deaf, blind, and with shunted hydrocephalus. The patient’s mother (III-6) aged 35 years is phenotypically healthy, is not blood related to the father, and is not from the same region. After taking a detailed family history, the mother mentioned a similar presentation of disseminated BCG infection leading to early infantile death in one of her own cousins **(** **Figure 1A**: III-11). There is a positive family history of recurrent adenitis among members of the patient’s paternal family including his paternal aunt (III-1) and paternal cousin (IV-2) **(** **Figure 1A** **)**.

## 3 Investigations

To investigate the patient’s abdominal complaints, an abdominal ultrasound scan was requested for the patient. This showed minimal intra-abdominal fluid and mesenteric and para-aortic lymph node adenitis, which was reported as highly suggestive of abdominal TB based on his previous history. The chest x-ray showed bilateral prominent hilar lymph nodes (**Figure 1C** **)**. The diagnosis of TB infection was further confirmed with PCRs using IS6110 and mtp40 primers on blood and sputum, respectively. Immunological investigation revealed elevated immunoglobulin G (IgG) for his age, 2,117 mg/dl (normal range: 380–1,400 mg/dl). IgG subclasses and specific antibody titers for protein and polysaccharide vaccines were not done, as they are unavailable in Sudan. The patient’s IgM and IgE levels were normal at 69 mg/dl (normal range: 37–225 mg/dl) and 53 IU/ml (normal range up to 155 IU/ml), respectively. His IgA level was low, 19 mg/dl (normal range: 30–250 mg/dl), dihydrorhodamine reduction (DHR) test by flow cytometer showed normal oxidative burst, and the patient’s complete blood count and lymphocyte subset are shown in **Tables 1** and **Table 2**, respectively.

**Table 1:** Hematological, LFT, RFT, and HIV findings in the patient’s blood sample.

Description^1^	Value	Normal range
TWBCs	14.8× 10^3^/µl	4.0- 7.0 × 10^3^/µl
RBCs	4.14× 10^6^/µl	4.05- 5.05 × 10^6^/µl
Hemoglobin	9.3 g/dl	11.0-17.0 g/dl
Hematocrit	27.0%	35.0%-47.0%
MCV	65.2 fl	79.0-92.0 fl
MCH	22.5 pg	26.0-33.5 pg
MCHC	34.4 g/dl	27.0-32.0 g/dl
Platelets	917× 10^3^/µl	150-450 × 10^3^/µl
Lym#	4.0× 10^3^/µl	0.7 - 3.1 ×10^3^/µl
NEUT#	7.6× 10^3^/µl	1.4 - 7 ×10^3^/µl
CRP	Normal	<5.0 mg/l
ESR	Normal	5-15 mm/h
*S. typhi* titer	1/20	<1/80
*S. paratyphi* titer	1/40	<1/80
LFT/RFT	Normal	7- 20 mg/dl
HIV	Negative	Negative

^1^CRP, C-reactive protein; ESR, erythrocyte sedimentation rate; MCV, mean corpuscular volume; MCH, mean corpuscular hemoglobin; MCHC, mean corpuscular hemoglobin concentration; TWBCs, total white blood cells; Lym#, lymphocyte count; NEUT#, neutrophil count; RBCs, red blood cells; LFT/RFT, liver function test/renal function test, HIV, human immunodeficiency virus. All of the values are within the standard reference range.

**Table 2:** Patient lymphocyte subset flow cytometry results.

Description	%	Normal range	Absolute count	Normal range
CD3	74%	60-67	3,943	1,200-2,600
CD3/CD4	53%	31-47	2,870	650-1,500
CD4/CD8	20	18-35	1,047	370-1,100
B cell (CD19)	17.3	13-27	1,088	270-800
NK (CD16)	8.24	4-17	587	100-400

Primary immunodeficiency genetic testing performed on the NextSeq Illumina platform revealed two pathogenic homozygous variants in *IL12RB1* gene of our patient at Exon 9, c.913A>T (p. Lys305*). This *IL12RB1* mutation is associated with autosomal recessive MSMD, which is reported by Altare et al. (11). This sequence change creates a premature translational stop signal (p.Lys305*) in the *IL12RB1* gene, which is expected to result in an absent or disrupted protein product (11). Such loss-of-function mutations in *IL12RB1* are pathogenic (PMID: 9603733, 12591909). The patient’s variant was not present in population databases (gnomAD no frequency), but this premature translational stop signal was observed in individuals with clinical features of MSMD (PMID: 9603732; Invitae). ClinVar also contained an entry for this variant (Variation ID: 488832). The father’s genotype showed the same homozygous pathogenic variants in *IL12RB1* Exon 9, c.913A>T (p. Lys305*), and interestingly, the mother, who is not blood related to the father, is a carrier for the same mutation. The patient is now on anti-TB drugs and planned to continue treatment for 2 years and then for bone marrow transplant.

## 4 Discussion

Disseminated BCG infection is the most serious complication of BCG vaccination. Fatal infection has occurred at a rate of 0.06–1.56 cases per million doses; these deaths occurred primarily among immunocompromised people (12, 13). The management of disseminated BCG infection should be prolonged for up to 24–36 months with a pyrazinamide-free 4-drug regimen (rifampicin, isoniazid, ethambutol, and levofloxacin ± clarithromycin). Then, a prophylactic anti-TB regimen with two drugs should be continued until complete immunological reconstitution after hematopoietic stem cell transplantation is achieved, which is found curative in MSMD cases (14).

The patient has *IL12RB1* Exon 9, c.913A>T (p.Lys305*) mutation, which results in the replacement of a lysine (K) by a stop codon (X) at amino acid position 305 and is referred to as K305X. This defect is associated with autosomal recessive MSMD, and it disrupts the coding region upstream of the transmembrane segment, preventing the receptor from being expressed on the surface of T lymphocytes and natural killer (NK) cells (11). The proband acquired a homozygous mutation from his parents. This is interesting, as the father is homozygous and affected by the disease, while the mother is a carrier of the same mutation despite the fact that there is no direct blood relation between the parents (**Figure 1A** **)**.

The patient’s homozygous mutation and MSMD phenotype could be explained by the high incidence of consanguinity and intermarriages in Sudan (15). The mother’s mutation could be due to an uncovered founder mutation in the Sudanese population (16). MSMD is considered a rare condition, and the population frequency of mutations leading to MSMD and other primary immunodeficiencies is not known in the Sudanese population. These mutations could be as frequent as cystic fibrosis (CF) pathogenic alleles in Caucasian populations where the CF phenotype penetrance occurs only when homozygous or compound heterozygous inheritance is exhibited. In such cases, consanguinity would enhance the occurrence of homozygosity and phenotype penetrance (16). Performing Sanger’s sequencing Whole‐Exome Sequencing on *IL12RB1* deficiency population would help in confirming gene variants in the Sudanese population. On the other hand, a haplotype search using Single Nucleotide Polymorphism array or WES on next-generation sequencing (NGS) followed by homozygosity mapping would reveal shared regions of homozygosity in the parents (17, 18).

Due to the unavailability of genetic testing in Sudan, a definitive diagnosis of MSMD remains challenging, and there are no data on the prevalence of MSMD in Sudan. The challenge to identify this phenotype increases in countries with prevalent endemic TB and the need for early BCG vaccination coupled with poor health systems and limited resources. Over the past 2 years, the Pediatric Immunology and Pediatric Tropical and Infectious Diseases units at the Tropical Diseases Hospital, Omdurman, Sudan, have encountered 44 patients with disseminated BCGosis (unpublished data). In this group of patients, a diagnosis of severe combined immunodeficiency (SCID) was made in 21 patients, chronic granulomatous disease (CGD) in eight patients, and MSMD in 15. These diagnoses were reached using the combination of clinical picture and immunological lab parameters. Only two out of the 15 patients suspected with MSMD had a confirmed diagnosis through genetic testing. Poor awareness regarding these conditions delayed the diagnosis of the father for many years (40 years old), which led to him being adversely affected with several TB infections throughout his life. The case of the father reflects the natural history of delayed diagnosis and treatment of patients with *IL12RB1* mutations.

Although no genetic testing was performed in other members of the family, it was found that many members were affected by recurrent or disseminated TB including the mother’s maternal cousin (III-11) who died at age 2 because of recurrent TB infections **(** **Figure 1A**). There was a similar clinical presentation with recurrent TB infections in the patient’s paternal aunt (III-1) and the patient’s paternal cousin (IV-2). This family pedigree also shows six first-degree consanguineous marriages that increase the possibility of occurrence of autosomal recessive conditions such as autosomal recessive MSMD. Unfortunately, we were only able to perform MSMD genetic testing on the patient and his parents. Other affected MSMD individuals were suspected by virtue of the history of recurrent TB infections.

Sudan is an East African country with complex genetic and population structures. This complexity stemmed from the linguistic and cultural differences between its ethnic groups acting in parallel with other, sometimes opposing, population genetic forces, e.g., consanguinity, admixture, and migration. For instance, 67% of marriages in some parts of the country are consanguineous (15, 19).

The frequency of the carrier state for MSMD causing mutations in the Sudanese population needs to be investigated to prevent their deleterious effects. This family’s offspring had a 50% chance of being diseased. Patients with MSMD typically present with BCG infections or environmental non-tuberculous mycobacteria (NTM) (3). Patients with MSMD are also susceptible to other intracellular pathogens, such as extraintestinal infection with typhoid, non-typhoid *Salmonella*, and mucocutaneous infections (5, 20). Here, the patient had a typical presentation with BCGitis after he received the BCG vaccine, but he was not investigated properly for disseminated BCGosis, and he received a short treatment regimen including pyrazinamide against which *Mycobacterium bovis* is naturally resistant (3).

Also, the patient had oral thrush that was due to candidiasis. This is consistent with studies that found that mucocutaneous candidiasis is present in approximately 25% of patients with *IL12RB1* mutation (10, 20, 21). This is likely due to impaired IL-23-dependent IL-17 immunity. The *IL12RB1* gene encodes the IL-12 receptor beta 1 subunit, which forms a heterodimeric receptor with the IL-12 receptor alpha 1 subunit. This receptor is responsible for the transduction of signals from the cytokines IL-12 and IL-23 (22). Impairment of the *IL12RB1* gene thus results in decreased signaling through the IL-12/IL-23 receptor complex, potentially impacting the immune function.

Limited resources prevent identifying whether the disseminated MSMD disease is due to *Mycobacterium tuberculosis* infection or reactivated latent infection with *M. bovis* infection acquired from the BCG vaccine, as the patient received a non-sufficient regimen for BCG infection in his early life. This is consistent with several studies that mentioned that before a diagnosis of MSMD, acquired and inherited immunodeficiencies that confer a predisposition to mycobacterial diseases must be excluded first (3, 23, 24).

Finally, it is important to note the importance of early diagnosis of such patients. Our patient was not investigated for possible immunodeficiency despite TB recurrences and a highly suggestive family history. This reflects the lack of awareness regarding conditions that result from genetic susceptibility to infection such as MSMD and other inborn errors of immunity. To ensure the best possible outcomes for this patient and the family, he will be under the care and close follow-up of the Pediatric Immunology and the Pediatric Tropical and Infectious Diseases units.

## 5 Conclusion

Diagnosing *IL12RB1* mutations should be considered in patients with recurrent, severe, or persistent TB infection especially after obtaining the BCG vaccination. BCGosis is another indicator of MSMD. Taking a detailed infection, immunization, and family history together with conducting immunological and genetic tests will identify MSMD early enough to avoid complications from the disease. Awareness programs regarding the identification of MSMD and other inborn errors of immunity are needed to improve patient outcomes and to enhance better care for such patients.

## Data availability statement

The original contributions presented in the study are included in the article/supplementary material. Further inquiries can be directed to the corresponding author.

## Ethics statement

The studies involving human participants were reviewed and approved by Tropical Diseases Teaching Hospital. Written informed consent to participate in this study was provided by the participants’ legal guardian/next of kin. Written informed consent was obtained from the minor(s)’ legal guardian/next of kin for the publication of any potentially identifiable images or data included in this article.

## Author contributions

OA, MuA, and NE cared the patient, performed medical examinations, and approved the study. AM, OA, and YA took the lead in writing the manuscript and family pedigree constriction. OS, AM, RH, HM, MH, MoA, and SA revised and wrote the manuscript. All authors reviewed the manuscript and provided critical feedback and agreed on the final manuscript. All authors contributed to the article and approved the submitted version.
